# Nirsevimab Prophylaxis and Respiratory Syncytial Virus Hospitalizations Among Infants

**DOI:** 10.1001/jamanetworkopen.2025.44679

**Published:** 2025-11-20

**Authors:** Enrico Cocchi, Silvia Bloise, Aurora Lorefice, Sara Zannoni, Benedetta Pellegrini, Francesco Saverio Morlupo, Beatrice Scarpellini, Melodie O. Aricò, Francesco Accomando, Gina Ancora, Gianluca Vergine, Enrico Valletta, Marcello Stella, Federico Marchetti

**Affiliations:** 1 Department of Medical and Surgical Sciences (DIMEC), Alma Mater Studiorum–University of Bologna, Bologna, Italy; 2 Neonatal Intensive Care Unit, Ravenna Hospital, AUSL Romagna, Ravenna, Italy; 3 Department of Precision Medicine and Genomics, Columbia University, New York, New York; 4 Pediatric Unit, Ravenna Hospital, AUSL Romagna, Ravenna, Italy; 5 Pediatric Residency Program, Alma Mater Studiorum–University of Bologna, Bologna, Italy; 6 Pediatric Residency Program, University of Ferrara, Ferrara, Italy; 7 Pediatric Unit, Rimini Hospital, AUSL Romagna, Rimini, Italy; 8 Pediatric Unit, Forli Hospital, AUSL Romagna, Forli, Italy; 9 Neonatal Intensive Care Unit, Rimini Hospital, AUSL Romagna, Rimini, Italy; 10 Neonatal Intensive Care and Pediatric Unit, Bufalini Hospital, AUSL Romagna, Cesena, Italy

## Abstract

**Importance:**

Respiratory syncytial virus (RSV) is the leading cause of infant hospitalizations, and preterm infants and those with older siblings are at increased risk. Despite recommendations for nirsevimab prophylaxis, data on outcomes in high-risk infants are lacking.

**Objective:**

To assess the association of nirsevimab prophylaxis with RSV-related lower respiratory tract infection (LRTI) hospitalization risk and in-hospital severity among infants, overall and by high-risk groups.

**Design, Setting, and Participants:**

This retrospective multicenter cohort study comparing RSV seasons before (April 1, 2023, to March 31, 2024) and after (April 1, 2024, to March 31, 2025) universal nirsevimab prophylaxis implementation included all live births from 5 neonatal hospitals serving the Italian provinces of Ravenna, Faenza, Forlì, Cesena, and Rimini with no exclusions based on gestational age or comorbidities. Participants were followed up from hospital discharge until first RSV hospitalization, first birthday, or season end, with time-to-event analysis. Data from centralized electronic medical records included demographics, nirsevimab administration, and polymerase chain reaction–confirmed RSV.

**Exposure:**

Nirsevimab prophylaxis vs no prophylaxis.

**Main Outcomes and Measures:**

The primary outcome was RSV-associated hospitalization in the first year of life. The secondary outcome was LRTI severity measures—hospital length of stay, high-flow nasal cannula (HFNC) use, and intensive care unit (ICU) admission. Hierarchical Cox proportional hazards regression models (with health care center as a random effect) were used to adjust for seasonality and relevant covariates, with sensitivity analyses using multiple models and non-RSV LRTI comparisons.

**Results:**

Among 13 624 newborns (mean [SD] gestational age of 39.4 [1.8] weeks; 51.4% male, 4.8% preterm, and 49.5% with older siblings), nirsevimab prophylaxis achieved 79.2% coverage of the study population. Among 292 infants hospitalized with RSV LRTI (2.1%), fewer were in the postnirsevimab than prenirsevimab season group (72 [24.7%] vs 220 [75.3%]; *P* < .001), with a population-level reduction in hospitalization hazard (hazard ratio [HR], 0.32; 95% CI, 0.25-0.44; *P* < .001). In a separate within-month analysis comparing infants born in the same calendar month and therefore at similar baseline RSV risk, nirsevimab prophylaxis was associated with a lower hazard of RSV hospitalization (HR, 0.11; 95% CI, 0.06-0.21; *P* < .001). Prematurity (HR, 2.93; 95% CI, 2.11-4.07; *P* < .001) and living with older siblings (HR, 4.57; 95% CI, 4.15-5.03; *P* < .001) remained associated with higher hospitalization risk among infants who received prophylaxis. Among hospitalized infants, nirsevimab was associated with reduced HFNC use (OR, 0.33; 95% CI, 0.11-0.97; *P* = .04) but not with shorter stays (incidence rate ratio, 0.81; 95% CI, 0.63-1.03; *P* = .09).

**Conclusions and Relevance:**

In this multicenter cohort study, nirsevimab prophylaxis was associated with substantially lower RSV hospitalization risk and reduced in-hospital RSV severity, supporting its implementation as a public health strategy. However, the persistent risk associated with prematurity and household RSV exposure suggests a need for supplemental approaches to optimize RSV prevention in high-risk infants.

## Key Points

**Question:**

Is nirsevimab prophylaxis associated with respiratory syncytial virus (RSV)–related hospitalization risk among infants, particularly in high-risk groups?

**Findings:**

In this cohort study of 13 624 infants in Italy, nirsevimab population coverage of 79% was associated with a significantly lower population-level and individual-level hazard of RSV hospitalization.

**Meaning:**

These findings suggest that nirsevimab prophylaxis is associated with substantially lower RSV hospitalization risk, but targeted supplemental strategies may be needed for high-risk infants to further reduce RSV burden.

## Teaser

This cohort study examines whether nirsevimab prophylaxis administered to newborns is associated with risk of hospitalization for and clinical severity of respiratory syncytial virus (RSV) in the first year of life.

## Introduction

Lower respiratory tract infections (LRTIs) are the leading cause of hospital admission in infants, contributing to a significant seasonal health care burden.^1,2,3^ The primary etiologic agent is respiratory syncytial virus (RSV),^4^ which has also been associated with long-term respiratory sequelae, including recurrent wheezing and asthma,^5^ emphasizing the urgent need for effective preventive strategies.^6^

Preterm infants are particularly susceptible to RSV because of immature immune responses.^7,8,9^ In addition, household transmission is a major driver of infant RSV, and prior studies consistently demonstrate higher risk with larger household size and the presence of older siblings.^8,9^ To date, no specific antiviral therapy has demonstrated efficacy, and management mainly relies on respiratory support.^10^ Historically, palivizumab—a monoclonal antibody targeting RSV—has been used for prophylaxis in high-risk infants.^11,12^ However, its broader application is limited by restrictive eligibility criteria, high cost, and logistic challenges, which have hindered widespread implementation.^13^

In November 2022, the European Commission approved nirsevimab, a long-acting monoclonal antibody targeting the RSV fusion protein.^14,15^ Unlike palivizumab, nirsevimab offers the advantage of single-dose administration and has broader applicability, potentially transforming RSV prevention strategies.^16^ Both clinical trials and early data have demonstrated nirsevimab’s efficacy in reducing RSV hospitalization risk.^17,18,19,20,21,22,23^

Italian national guidelines now recommend universal prophylaxis for all infants born during the yearly RSV epidemic period (September 1 to March 31) as well as for children previously eligible for palivizumab.^24,25^ Despite these advances, evidence on the impact of nirsevimab in routine clinical practice remains limited.

A critical question is whether nirsevimab’s protective effect is consistent across gestational age groups or whether preterm infants experience attenuated benefits given their inherent susceptibility. Of note, current evidence on this interaction is largely extrapolated from clinical trials,^26^ with no studies to our knowledge available to confirm the robustness of the evidence. A prior study by our group, which included only hospitalized infants, found that preterm infants remained at higher risk for severe RSV outcomes despite receiving prophylaxis.^27^ Furthermore, to our knowledge, the role of household exposure—particularly the presence of older siblings—in modifying RSV hospitalization risk under prophylaxis remains totally unexplored.

This multicenter cohort study addressed these critical knowledge gaps by leveraging comprehensive regional health data from 5 neonatal centers across 2 consecutive RSV epidemic seasons in Italy. Our primary objective was to quantify the association of nirsevimab prophylaxis with RSV-related hospitalization and RSV severity during the first winter of life. Additionally, we aimed to explore the independent contributions of prematurity and household exposure (older siblings) to RSV hospitalization risk and disease severity.

## Methods

### Study Design and Population

We conducted a retrospective multicenter cohort study to assess the association of nirsevimab prophylaxis with RSV-related hospitalizations during the first winter of life. The study included all live births from 5 neonatal centers in the Italian provinces of Forlì, Cesena, Rimini, Faenza, and Ravenna, covering 2 consecutive RSV epidemic seasons: April 1, 2023, to March 31, 2024 (prenirsevimab), and April 1, 2024, to March 31, 2025 (postnirsevimab). Written parental informed consent was obtained for all hospitalized infants, and data were collected for live births as per the protocol approved by the Comitato Etico Della Romagna. The study was reported in accordance with the Strengthening the Reporting of Observational Studies in Epidemiology (STROBE) reporting guideline for cohort studies.

Only infant residents within the regional catchment area were included, to ensure complete follow-up and standardized access to prophylaxis and care. Infants were followed up from birth until the earliest of the following: (1) first RSV-related hospitalization, (2) April 1 of the next year (seasonal cutoff), or (3) their first birthday. LRTI was defined using discharge diagnoses that included any code for lower respiratory infection—pneumonia, acute bronchitis, acute bronchiolitis, or unspecified acute LRTI. Nirsevimab was administered to all newborns, following informed parental consent, before hospital discharge after birth. Data were extracted through passive surveillance from centralized electronic medical records, which capture all births and inpatient admissions, and included demographic information, prophylaxis administration, clinical variables at admission, and virologic confirmation of RSV infection by polymerase chain reaction using a nasopharyngeal swab sample. Hospitalizations were classified as RSV-related if the swab sample tested positive for RSV at admission and as non-RSV otherwise. Infants who received palivizumab were included in the prenirsevimab group, as this reflected standard of care prior to nirsevimab introduction. Electronic medical record infrastructure, admission workflows, and diagnostic coding were stable across the study period; between-hospital heterogeneity was modeled with a random intercept. As a negative-control outcome, we analyzed non-RSV LRTI hospitalizations to detect secular changes in care-seeking or admission thresholds between epochs (ie, differential health care use unrelated to nirsevimab).

### Exposure and Outcomes

The primary exposure was receipt of nirsevimab prophylaxis. The primary outcome was RSV-related hospitalization during the first winter of life. Secondary outcomes assessing LRTI severity in hospitalized infants included length of stay, need for high-flow nasal cannula (HFNC), and intensive care unit (ICU) admission.

All models were adjusted for known relevant baseline covariates selected a priori based on published risk factors and clinical plausibility: prematurity status (<37 weeks of gestational age), sex, presence of older siblings, and seasonality.^8,9^ We defined presence of older siblings as at least 1 coresident child under 15 years of age who was older than the index infant at birth. Due to data constraints, we could not further stratify sibling age or retrieve information regarding comorbidities and prenatal or postnatal tobacco exposure. To account for potential geographic and center-specific variability, a random intercept was included for health care center. Non–data-driven covariate selection was performed.

### Statistical Analysis

We used hierarchical Cox proportional hazards regression models, with the event defined as RSV-related LRTI hospitalization. To avoid underestimation of time at risk, especially in preterm infants with prolonged birth hospitalizations, follow-up was defined starting from hospital discharge after birth. Time to event was calculated from this discharge date to the event (RSV-related hospitalization) or censoring. All models were adjusted for nirsevimab prophylaxis, prematurity, sex, and the presence of older siblings in the household. Given the pronounced seasonality of RSV (and nirsevimab uptake), we stratified the Cox proportional hazards regression model by month of birth to estimate the within-month, individual-level association, acknowledging that baseline risk varies markedly across months. To examine whether the association of nirsevimab with RSV-related LRTI hospitalization varied by gestational age, we included an interaction term between nirsevimab prophylaxis and prematurity status (nirsevimab × prematurity). We also tested a nirsevimab × siblings interaction as a specificity check for effect modification, given no a priori biological rationale for siblings to alter pharmacologic response.

To account for potential geographic variability, we included random effects for health care center in all models. To capture the population impact of the nirsevimab prophylaxis rollout, we also modeled epoch (2023-2024 vs 2024-2025) as the primary exposure in a Cox proportional hazards regression model, with a hospital random intercept. The epoch model estimated a population-level rollout association (population hazard ratio [HR]); the month-stratified model estimated the within-month individual association (individual HR). Cases with missing data in any model covariate were excluded. Results are presented as HRs with corresponding 95% CIs and *P* values. For visual representation of the findings, unadjusted Kaplan-Meier survival curves were generated for patients receiving and not receiving nirsevimab prophylaxis using the same outcome and time-at-risk definitions as in the Cox proportional hazards regression models, stratified by prematurity status.

For secondary outcomes, we used the same covariates and random effects as in the primary analysis. Given the distribution, length of stay was modeled with a hierarchical Poisson regression using a log link, while HFNC use and ICU admission were each analyzed via hierarchical logistic regression. For the Poisson length-of-stay model, we assessed overdispersion using the Pearson χ^2^ diagnostics. If significant overdispersion was present (φ > 1.2), we refit a negative binomial mixed model and, as a sensitivity analysis, a Poisson model with an observation-level random effect. Results are reported as incidence rate ratios (IRRs) for length of stay and odds ratios (ORs) for HFNC and ICU, all with 95% CIs and *P* values.

To assess the proportional hazards assumption of the mixed-effects Cox proportional hazards regression model, we applied the scaled Schoenfeld residual test, evaluating the correlation between the residuals and time for each covariate, visually inspecting Schoenfeld residual plots to identify potential time-dependent patterns (eFigure in Supplement 1). A global test was also performed to assess the assumption across the entire model.

To ensure the robustness of our findings, we conducted multiple sensitivity analyses on the primary outcome. First, we repeated the primary models using non-RSV LRTI hospitalizations, for which no association with nirsevimab was expected. We further tested consistency across model structures by replicating the main Cox proportional hazards regression analysis with logistic regression. Additionally, to validate the month-of-birth stratification, we replaced it with a restricted cubic spline of day of year at birth (Akaike information criterion–selected knots) and varied the knot number from 4 to 24 and the period length (resulting in resolutions varying from 15 to 90 days). Finally, we refit the Cox proportional hazards regression model restricted to RSV-season births after confirming stable coverage across months.

All analyses were performed using survival, coxme, glmer, and ggplot2 packages of R, version 4.4.2 (R Project for Statistical Computing). Two-sided *P* < .05 was considered significant.

## Results

**Figure 1.**
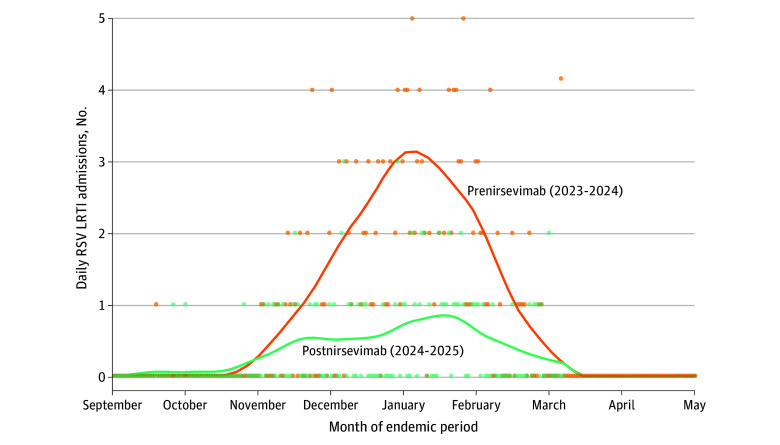
Daily Respiratory Syncytial Virus Lower Respiratory Tract Infection (RSV LRTI) Hospital Admissions Before and After Nirsevimab Introduction Scatter points represent the number of RSV LRTI admissions on each day over the epidemic period (September 1 to March 31) for the prenirsevimab and postnirsevimab seasons. Low-degree polynomials in localized neighborhoods and locally estimated scatterplot smoothing were used to produce smooth curves (indicated by solid lines) that illustrate the overall daily admission trends for each season.

A total of 13 624 eligible live births were recorded across the 5 participating hospitals, of which 6618 (48.6%) were female and 7006 (51.4%) were male, with mean (SD) gestational age of 39.4 (1.8) weeks. Of the included participants, 6738 (49.5%) had an older sibling within the household and 660 (4.8%) were born preterm. A total of 6940 births (50.9%) occurred during the prenirsevimab season and 6684 (49.1%) in the postnirsevimab season. Nirsevimab prophylaxis was administered to 79.2% of the eligible newborn population after September 1, 2024, reflecting the program’s broad implementation and high uptake. In all, 292 infants (2.1%) experienced RSV-related hospitalization: 220 (75.3%) in the prenirsevimab season and 72 (24.7%) in the postnirsevimab season (*P* < .001). Among the 72 hospitalizations in the postnirsevimab season, 54 (75.0%) occurred in infants who had not received prophylaxis. Non-RSV LRTI hospitalizations remained stable in the prenirsevimab and postnirsevimab seasons: 46 of 80 (57.5%) and 34 of 80 (42.5%), respectively (*P* = .29). Figure 1 presents daily hospital admissions for RSV LRTI in the cohort, grouped by epidemic season (prenirsevimab and postnirsevimab periods). Premature infants made up a larger percentage of the RSV-related LRTI hospitalizations in the postnirsevimab epoch compared with the prenirsevimab epoch, rising from 25 of 220 (11.4%) to 11 of 72 (15.3%) (*P* < .001). Baseline characteristics of the cohort are provided in the Table.

**Table.** Baseline Characteristics by Nirsevimab Status, Hospitalization Type, and Epoch^a^

Characteristic	Overall (N = 13 624 [100])^c^	Nirsevimab		RSV LRTI		Non–RSV LRTI		2024-2025 Season^b^	
		No. (%) (n = 3145 [23.1])	*P* value^d^	No. (%) (n = 292 [2.1])	*P* value^d^	No. (%) (n = 80 [0.6])	*P* value^d^	No. (%) (n = 6684 [49.1])	*P* value^d^
Sex									
Female	6618 (48.6)	1532 (48.7)	.88	134 (45.9)	.39	39 (48.8)	.49	3251 (48.6)	.89
Male	7006 (51.4)	1613 (51.3)		158 (54.1)		41 (51.2)		3433 (51.4)	
RSV LRTI	292 (2.1)	18 (0.6)	<.001	NA	NA	NA	NA	72 (1.1)	<.001
Non–RSV LRTI	80 (0.6)	15 (0.5)	.43	NA	NA	NA	NA	34 (0.5)	.33
Older sibling in household	6738 (49.5)	1542 (49.0)	.60	235 (80.5)	<.001	69 (84.9)	<.001	3350 (50.1)	.99
Preterm	660 (4.8)	202 (6.4)	<.001	36 (12.3)	<.001	13 (16.2)	<.001	290 (4.3)	.007
Length of hospital stay, median (IQR), d	4 (2-6)	4 (2-6)	.59	4 (3-6)	NA	4 (2-7)	NA	4 (2-6)	.72
HFNC	214 (1.6)	14 (0.4)	<.001	168 (57.7)	NA	46 (58.2)	NA	52 (0.8)	<.001
ICU	46 (0.3)	3 (0.1)	.01	42 (14.4)	NA	4 (5.0)	NA	7 (0.1)	<.001

Abbreviations: HFNC, high-flow nasal cannula; ICU, intensive care unit; LRTI, lower respiratory tract infection; NA, not applicable; RSV, respiratory syncytial virus.

^a^ Data are presented as number (percentage) of participants unless otherwise indicated.

^b^ The season column shows characteristics of infants in the postnirsevimab epoch (2024-2025).

^c^ The overall column includes all infants, whether they did or did not receive nirsevimab prophylaxis.

^d^ *P* values correspond to univariate comparisons between groups: nirsevimab (yes vs no), RSV LRTI (yes vs no), non-RSV LRTI (yes vs no), and season (2024-2025 [postnirsevimab] vs 2023-2024 [prenirsevimab]).

### Primary Outcome

#### Nirsevimab and RSV Hospitalization

Nirsevimab prophylaxis was associated with a reduction in the within-month individual hazard of RSV hospitalization (HR, 0.11; 95% CI, 0.06-0.21; *P* < .001). Logistic regression supported this result (OR, 0.11; 95% CI, 0.06-0.19; *P* < .001). No significant association with non-RSV hospitalizations was observed. All sensitivity analyses–restricted cubic spline models of day of year with 4 to 24 knots and a model restricted to the RSV season (with stable monthly coverage at 438 of 576 participants [76.0%] to 451 of 549 participants [82.1%]) produced materially unchanged HRs with overlapping 95% CIs.

At the population level, modeling epoch (2023-2024 vs 2024-2025) as the exposure was associated with a lower hazard of RSV hospitalization in 2024-2025 (HR, 0.32; 95% CI, 0.25-0.44; *P* < .001). This population HR was coherent with the individual HR given the estimated 79.2% effective nirsevimab coverage of the study population in 2024-2025 via the mixture identity, *HR_pop_* = *λ_post_(t*)/*λ_0_(t)* = (1 − *coverage*[1 − *HR_ind_*]), where *λ_0_*(*t*) represents the baseline hazard without prophylaxis; *HR_pop_*, the population-level epoch HR; *HR_ind_*, the individual (within-month) HR among infants receiving nirsevimab prophylaxis; and *coverage*, the effective coverage as the fraction of infant person-time protected in 2024-2025, which estimated *HR_pop_* as 1 − 0.79 × (1 − 0.11) = 0.30, closely matching the observed HR of 0.32.

#### Prematurity and RSV Hospitalization

**Figure 2.**
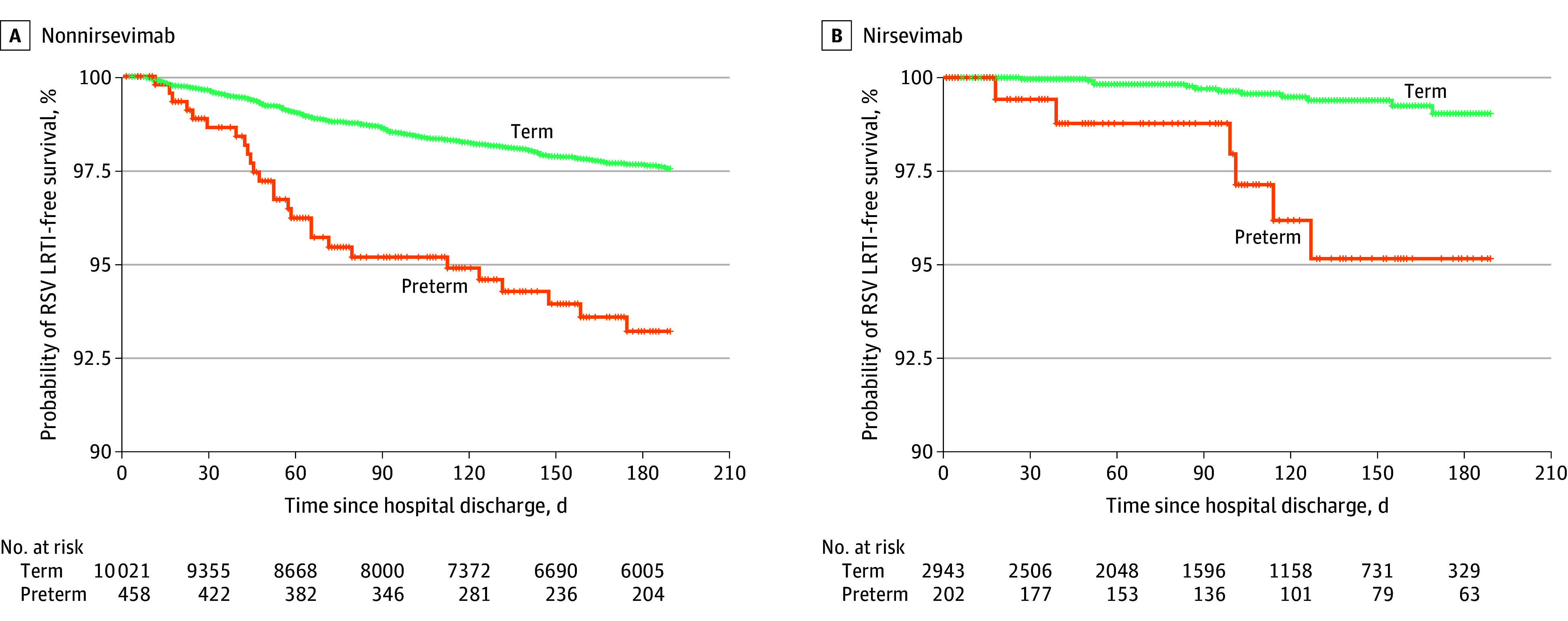
Kaplan-Meier Curves for Respiratory Syncytial Virus Lower Respiratory Tract Infection (RSV LRTI)–Free Survival Stratified by Gestational Age and Prophylaxis Status The probability of remaining free from RSV LRTI hospitalization over time for term (≥37 weeks’ gestation) and preterm (<37 weeks’ gestation) infants is shown. The follow-up (x-axis) is truncated at 180 days because nirsevimab prophylaxis began in September 2024 and by March 2025 this represented the maximum follow-up duration of any infant receiving prophylaxis. Tick marks denote censoring events.

Premature infants showed a substantially higher hazard of RSV hospitalization (HR, 2.93; 95% CI, 2.11-4.07; *P* < .001). Logistic regression supported this association (OR, 2.85; 95% CI, 1.91-4.28; *P* < .001). Prematurity was also associated with a markedly increased risk of non-RSV hospitalization (HR, 6.58; 95% CI, 3.58-12.11; *P* < .001). Population-level analysis (epoch model) yielded a prematurity association virtually identical to the primary model (HR, 2.96; 95% CI, 2.08-4.19; *P* < .001). Likewise, models using restricted cubic splines of day of year and the analysis restricted to RSV-season births produced materially identical estimates with overlapping 95% CIs. Kaplan-Meier curves stratified by gestational age and prophylaxis group are shown in Figure 2.

#### Interaction Between Nirsevimab and Prematurity

There was no interaction for nirsevimab × prematurity (HR, 1.58; 95% CI, 0.73-3.39; *P* = .19). The logistic model was concordant, and there was no association for non-RSV hospitalizations. Sensitivity analyses using restricted cubic splines of day of year and restricting to RSV-season births produced materially similar estimates with overlapping 95% CIs.

#### Older Siblings and RSV Hospitalization

Living with older siblings was associated with an increased hazard of RSV hospitalization (HR, 4.57; 95% CI, 4.15-5.03; *P* < .001), supported by the logistic model (OR, 4.71; 95% CI, 3.51-6.32; *P* < .001). The association remained for non-RSV LRTI (HR, 6.88; 95% CI, 3.64-13.01; *P* < .001). The population level (epoch) model showed consistent results (HR, 4.37; 95% CI, 3.27-5.84; *P* < .001). Sensitivity analyses using restricted cubic splines of day of year and restricting to RSV-season births produced materially similar estimates with overlapping 95% CIs.

#### Interaction Between Nirsevimab and Siblings

The nirsevimab × siblings interaction was not significant, with concordant nonsignificant findings in the secondary logistic model and in the non-RSV hospitalization analysis. Sensitivity analyses using restricted cubic splines of day of year and restricting to RSV-season births yielded consistent results.

#### Model Diagnostics

The HRs for prematurity and siblings were consistent across all models (month-stratified, epoch, spline, and RSV season only), supporting robustness of these associations. By contrast, the nirsevimab HR differed as expected: the epoch model estimated a population-level association, while the month-stratified model captured the individual-level association. The proportional hazards assumption was met, with no significant violations and residual plots within confidence limits.

### Secondary Outcomes

#### Length of Hospitalization

The hierarchical Poisson regression analysis revealed that prematurity was significantly associated with longer hospitalization (IRR, 1.33; 95% CI, 1.13-1.54; *P* < .001), whereas nirsevimab was not associated with shorter length of stay (IRR, 0.81; 95% CI, 0.63-1.03; *P* = .09). All other covariates had no associations. As resulting counts were significantly overdispersed (Pearson φ = 2.07), we refit a negative binomial mixed-effects model that yielded stable estimates. For robustness, a Poisson model with an observation-level random effect produced virtually identical IRRs and CIs.

#### HFNC Need

Among infants hospitalized with RSV LRTI, a hierarchical logistic regression showed that nirsevimab prophylaxis was associated with a significant reduction in HFNC need (OR, 0.33; 95% CI, 0.11-0.97; *P* = .04). Prematurity was not associated with increased HFNC use, and no other covariates had associations with HFNC need.

#### ICU Hospitalization

In the hierarchical logistic regression, no covariates were associated with ICU hospitalization. Although univariate testing showed a significant inverse association between nirsevimab and ICU hospitalization, it was not supported in the adjusted model.

## Discussion

In this multicenter cohort of 13 624 newborns, we found robust evidence that nirsevimab was associated with a significant reduction in RSV-related hospitalizations during the first winter of life. With 79.2% prophylaxis coverage of the study population, nirsevimab was linked to a 68% relative reduction in RSV hospitalization risk—closely mirroring efficacy reported in pivotal clinical trials and prior studies.^17,18,19,20,21,22,23^

Preterm birth and having older siblings within the household were found to be factors independently associated with RSV hospitalization even among infants who received nirsevimab, indicating that residual risk remains elevated in these high-risk groups.^28^ We found no evidence that siblings or prematurity significantly modified the nirsevimab outcome, supporting the interpretation that these risk factors capture intrinsic factors and environmental exposure intensity rather than altering monoclonal antibody pharmacodynamics.

Our findings suggest that nirsevimab prophylaxis is associated with a milder clinical course of RSV LRTI in hospitalized infants, as evidenced by a significant reduction in HFNC requirements. Although there were no associations with length of stay and ICU admission, point estimates favored protection and may have been underpowered due to the small number of treated infants, which produced a wide 95% CI and insufficient power once hospital-level clustering and covariate adjustment were taken into account. Overall, these findings suggest that even breakthrough RSV infections have a milder course in infants who have received nirsevimab prophylaxis, though larger studies are needed to clarify the impact on critical care utilization.

From a clinical perspective, universal nirsevimab prophylaxis was associated with markedly reduced RSV hospitalizations and a lower LRTI burden across the RSV season—a period that previously placed extraordinary strain on health care systems.^25,29^ However, the residual risk observed among preterm infants and those with older siblings suggests that nirsevimab alone may not fully address the heightened susceptibility associated with these factors. Collectively, these findings underscore the need for complementary public health interventions, such as different nirsevimab dosing regimens or maternal vaccination, and strengthened infection-control practices in households with high-risk infants to further alleviate the RSV disease burden.

### Limitations

This study has several limitations. Despite adjustment for prematurity, sex, and household exposure, residual confounding may persist due to unmeasured factors such as parental health literacy, comorbidities, or access to care. The short follow-up precluded assessment of longer-term outcomes. We anchored follow-up at birth discharge to avoid immortal time bias and modeled hospital clustering with random effects. Seasonality was controlled by month-of-birth strata and, in sensitivity analyses, by restricted cubic splines, RSV-season restriction, and a calendar-epoch model, but residual time-related confounding cannot be excluded. The sibling variable was binary and lacked age detail, and socioeconomic or household-level factors were unavailable. Such omissions may allow residual confounding. Hospitalizations outside the regional network were not captured, even if these events should be rare and nondifferential. In addition, while sensitivity analyses support robustness, they cannot fully exclude residual bias.

## Conclusions

This multicenter cohort of 13 624 newborns found that nirsevimab prophylaxis was associated with reduction of RSV-related hospitalizations and milder disease course. However, prematurity and household exposure remained significant independent risk factors, highlighting the necessity for additional, targeted prevention measures in these susceptible groups. Collectively, our results strongly endorse the integration of nirsevimab into routine immunization schedules and emphasize the need for continued surveillance to maximize its benefits on infant health outcomes.

## Notes

Supplement 1. **eFigure.** Proportional-Hazards Diagnostics (Schoenfeld Residuals) for the Primary Multivariate Cox Model

Supplement 2.
Data Sharing Statement
