# Supplementary material for: Nirsevimab Prophylaxis and Respiratory Syncytial Virus Hospitalizations Among Infants
Source: JAMA Netw Open. 2025 Nov 20;8(11):e2544679. doi: 10.1001/jamanetworkopen.2025.44679 (PMC12635873; doi:10.1001/jamanetworkopen.2025.44679)
Supplement: Supplement 1. — eFigure. Proportional-Hazards Diagnostics (Schoenfeld Residuals) for the Primary Multivariate Cox Model [file jamanetwopen-e2544679-s001.pdf]

Supplementary Online Content

Cocchi E, Bloise S, Lorefice A, et al. Nirsevimab prophylaxis and respiratory syncytial virus hospitalizations among infants. *JAMA Netw Open*. 2025;8(11):e2544679. doi:10.1001/jamanetworkopen.2025.44679

eFigure. Proportional-Hazards Diagnostics (Schoenfeld Residuals) for the Primary Multivariate Cox Model

This supplementary material has been provided by the authors to give readers additional information about their work.

© 2025 Cocchi E et al. *JAMA Network Open*.

eFigure. Proportional-hazards diagnostics (Schoenfeld residuals) for the primary multivariate Cox model.

Panels show scaled Schoenfeld residuals plotted against analysis time (days from birth-discharge) for each covariate in the model. Points are individual residuals; the solid line is a loess smooth; dashed lines are ±2 SE (pointwise 95% bands). A horizontal line at zero indicates the proportional-hazards (PH) expectation; systematic departures suggest time-varying associations.

PH tests: nirsevimab p=0.065, preterm p=0.418, older siblings p=0.074, male sex p=0.987, nirsevimab×preterm p=0.929; global p=0.084.

No statistically significant departure from proportional hazards overall. Minor drifts for nirsevimab and older siblings are near-threshold but remain small and within uncertainty bands, indicating no material non-proportionality.

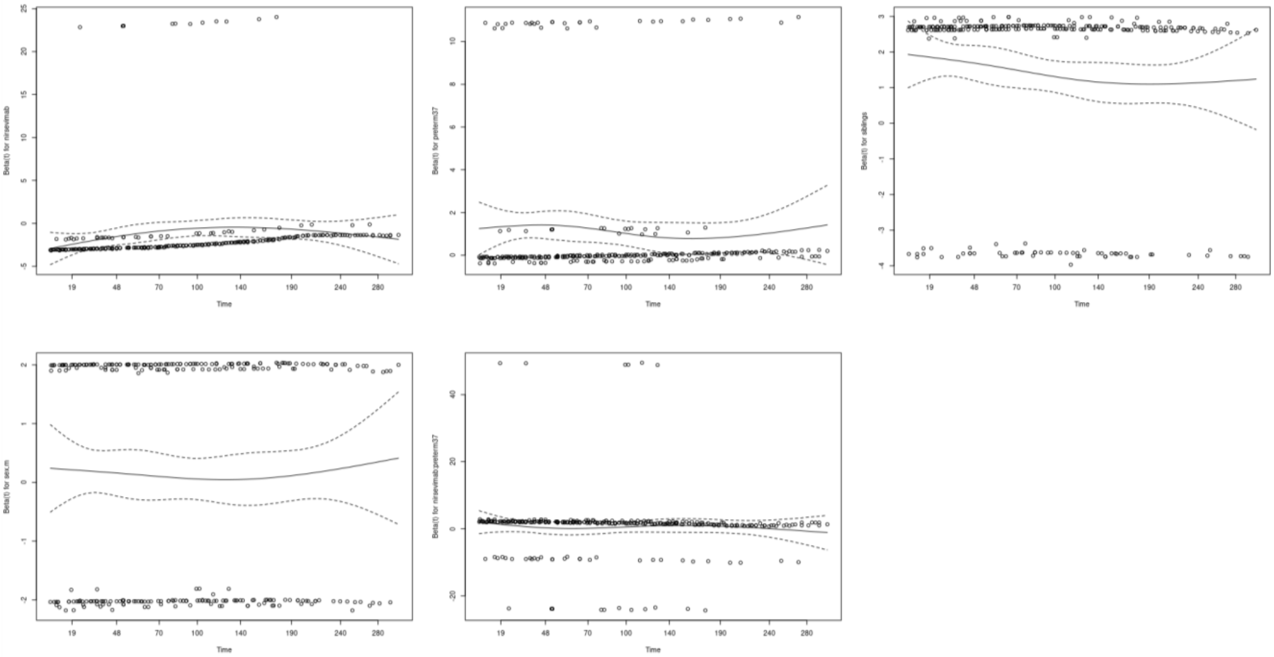

© 2025 Cocchi E et al. *JAMA Network Open*.
