# Supplementary material for: Nirsevimab Prophylaxis and Respiratory Syncytial Virus Hospitalizations Among Infants
Source: JAMA Netw Open. 2025 Nov 20;8(11):e2544679. doi: 10.1001/jamanetworkopen.2025.44679 (PMC12635873; doi:10.1001/jamanetworkopen.2025.44679)
Supplement: Supplement 2. — Data Sharing Statement [file jamanetwopen-e2544679-s002.pdf]

Data Sharing Statement

Cocchi. Nirsevimab Prophylaxis and Respiratory Syncytial Virus Hospitalizations Among Infants. *JAMA Netw Open*. Published November 20, 2025.
doi:10.1001/jamanetworkopen.2025.44679

Data
Data available: Yes
Data types: Deidentified participant data
How to access data: Completely anonymized data will be available to qualified academic investigators to replicate study results by reasonable request. Data transfer will be regulated by material transfer agreements.
When available: With publication

Supporting Documents
Document types: None

Additional Information
Who can access the data: Completely anonymized data will be available to qualified academic investigators to replicate study results by reasonable request. Data transfer will be regulated by material transfer agreements.
Types of analyses: Completely anonymized data will be available to qualified academic investigators to replicate study results by reasonable request. Data transfer will be regulated by material transfer agreements.
Mechanisms of data availability: Completely anonymized data will be available to qualified academic investigators to replicate study results by reasonable request. Data transfer will be regulated by material transfer agreements.
